# Adult ileocolic intussusception with lymphoma as the lead point: a case report

**DOI:** 10.1093/jscr/rjag401

**Published:** 2026-05-29

**Authors:** Nikita Satam, Monika Joshi, Nitish Jhawar

**Affiliations:** Department of Surgery, Apollo Hospital Navi Mumbai, Navi Mumbai, Maharashtra, India; Department of Surgery, Apollo Hospital Navi Mumbai, Navi Mumbai, Maharashtra, India; Department of Surgery, Apollo Hospital Navi Mumbai, Navi Mumbai, Maharashtra, India

**Keywords:** ileocolic intussusception, non-Hodgkin lymphoma, autism spectrum disorder, right hemicolectomy, bowel obstruction

## Abstract

Adult ileocolic intussusception is almost always associated with a pathological lead point. We present a 29-year-old male with autism spectrum disorder (ASD) who attended with a one-week history of colicky right iliac fossa pain and loose stools. Communication difficulties related to ASD delayed a clear clinical history. Abdominal ultrasonography demonstrated a 7.0 × 6.0 cm target sign in the subhepatic region. Contrast-enhanced computed tomography confirmed ileocolic intussusception extending to the hepatic flexure, with a heterogeneously enhancing 6.6 × 4 × 3.4 cm lead-point mass. Diagnostic laparoscopy was converted to open right hemicolectomy with ileocolic anastomosis. Histopathology revealed B-cell non-Hodgkin lymphoma, confirmed by immunohistochemistry (CD20+, BCL2+, BCL6+, MUM1+, and c-MYC+). The patient was referred for oncological management and was well at one-month review. This case highlights that adult intussusception caused by lymphoma can present insidiously, particularly in patients with impaired communication, and that early cross-sectional imaging and prompt surgical resection are essential.

## Introduction

Adult intussusception is rare, accounting for ~5% of all intussusceptions and 1%–5% of bowel obstructions [1, 2]. In contrast to the largely idiopathic paediatric condition, over 90% of adult cases harbour an identifiable pathological lead point, with neoplasia responsible for 60%–70% of cases [3]. Lymphoma is an important, though infrequently reported, cause. Adults typically present with non-specific symptoms including intermittent abdominal pain, nausea, and altered bowel habit, making timely diagnosis challenging [4]. These difficulties are compounded further in patients with autism spectrum disorder (ASD), where impaired verbal communication may obscure the clinical picture. Contrast-enhanced computed tomography (CT) is the gold-standard diagnostic modality, providing precise delineation of the intussusception and its lead point [5]. Surgical resection following oncological principles is the definitive treatment when malignancy is suspected [6].

## Case report

A 29-year-old male with ASD (BMI 28 kg/m^2^) presented with a one-week history of dull, colicky right iliac fossa pain radiating to the periumbilical region, 10–12 loose stools per day with yellowish mucus, and abdominal bloating. There was no haematochezia, melaena, fever, or weight loss. He was not on regular medications and had no family history of malignancy.

On examination he was haemodynamically stable. Abdominal examination revealed mild distension with right iliac fossa and periumbilical tenderness; no mass was palpable. Digital rectal examination was normal. Haemoglobin was 13.6 g/dl with normal leucocyte count and biochemistry.

**Figure 1:**
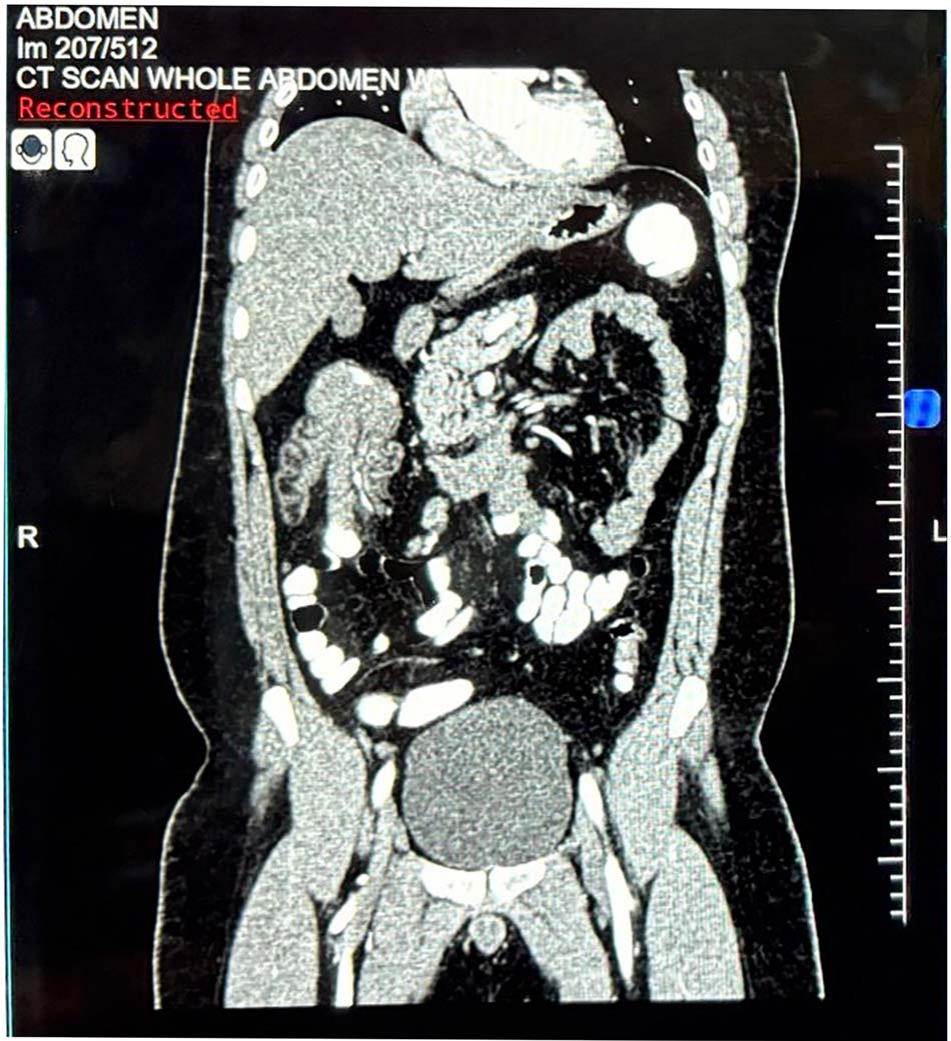
Coronal contrast-enhanced computed tomography of the abdomen. Coronal reconstruction demonstrating ileocolic intussusception extending to the hepatic flexure of the colon.

**Figure 2:**
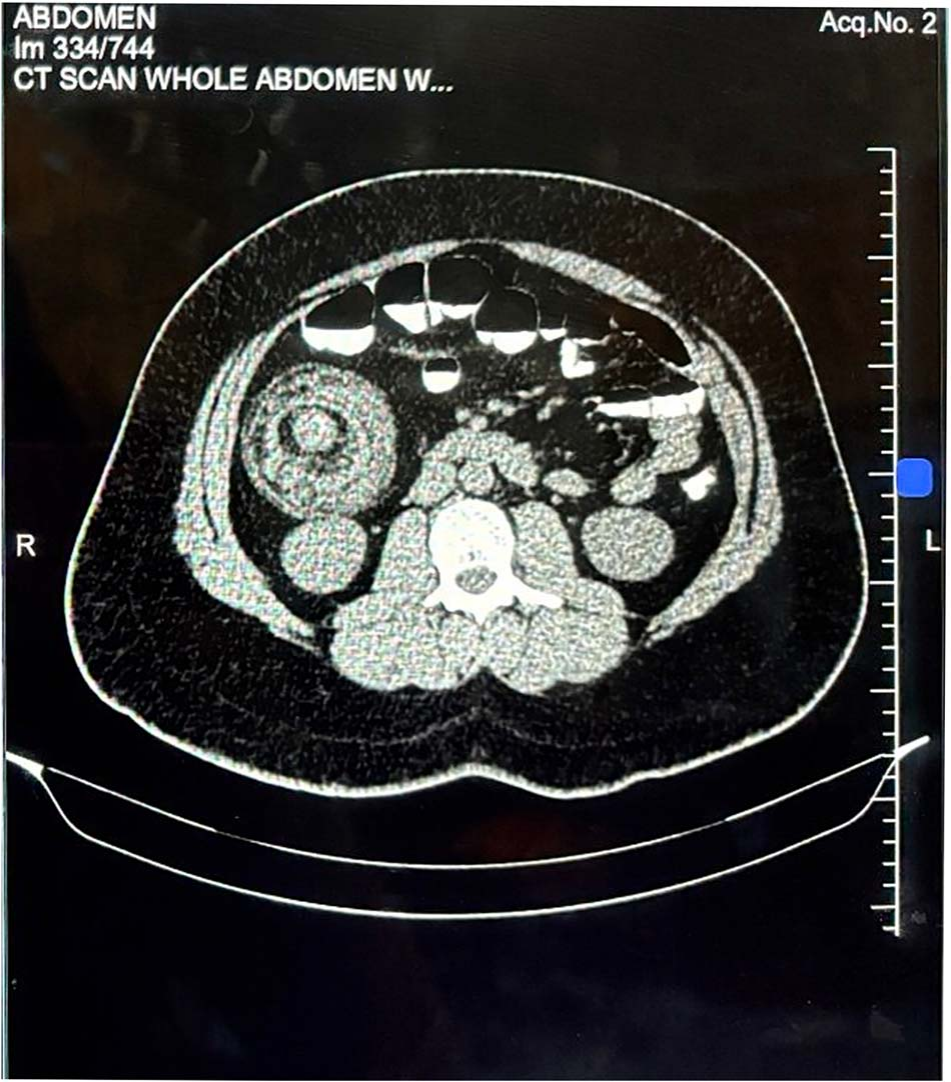
Axial contrast-enhanced computed tomography of the abdomen. Axial section demonstrating the characteristic "target sign" with a heterogeneously enhancing intraluminal mass at the lead point of the intussusception.

Abdominal ultrasonography demonstrated a 7.0 × 6.0 cm target sign in the subhepatic region involving the caecum and ascending colon to the hepatic flexure. Contrast-enhanced CT of the abdomen and pelvis confirmed ileocolic intussusception measuring 8.3 cm, with a heterogeneously enhancing 6.6 × 4 × 3.4 cm lead-point lesion (Figs 1 and 2).

**Figure 3:**
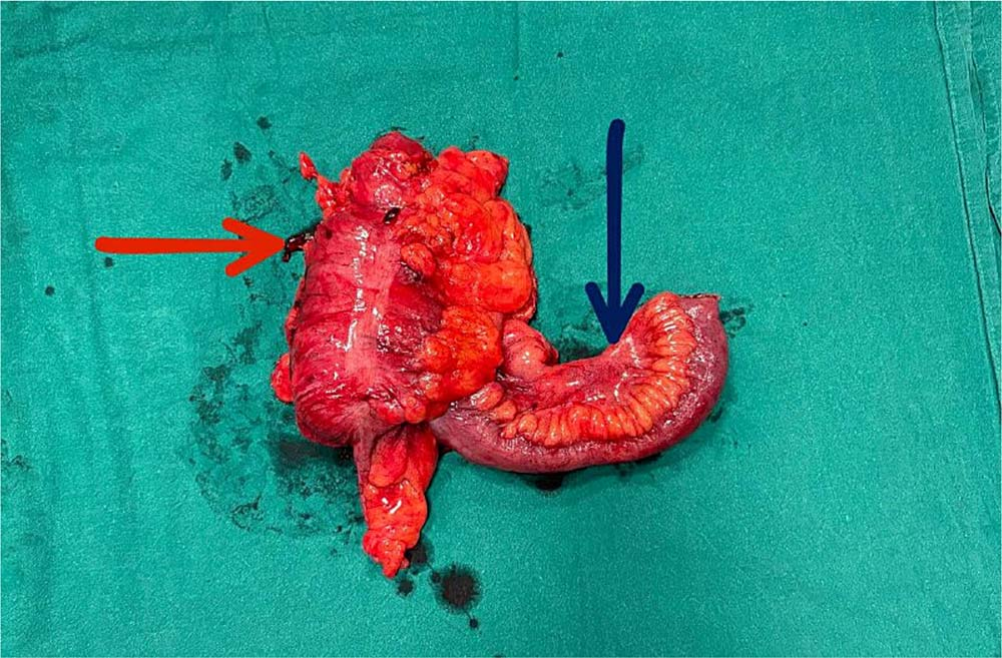
Gross specimen of the right hemicolectomy. The horizontal arrow indicates the ascending colon (intussuscipiens); the vertical arrow indicates the terminal ileum (intussusceptum) telescoping within it.

**Figure 4:**
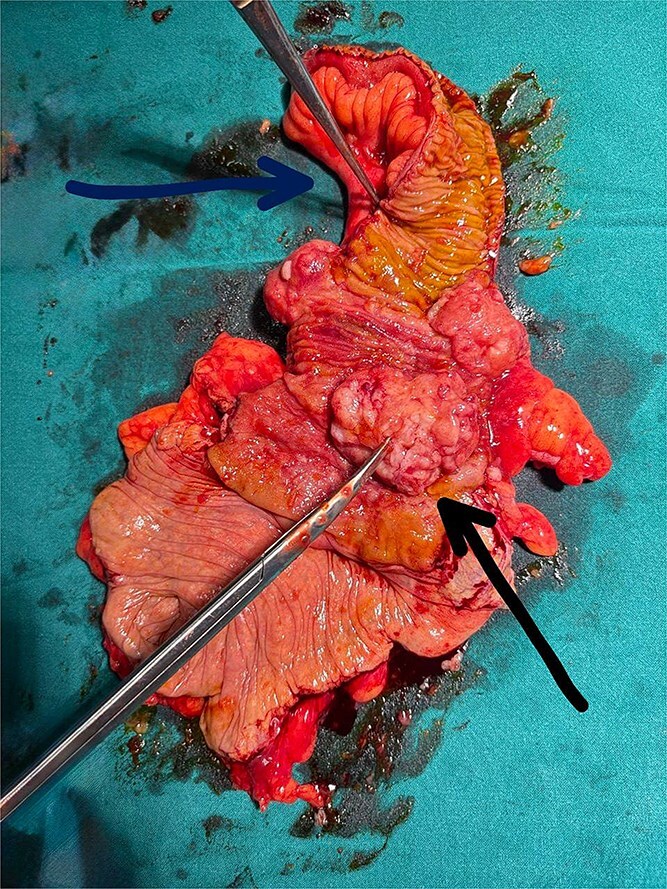
Cut-open gross specimen of the right hemicolectomy. The horizontal arrow indicates the opened bowel lumen demonstrating the intussuscepted mucosa; the diagonal arrow indicates the polypoid tumour at the ileocaecal junction serving as the lead point of the intussusception.

Diagnostic laparoscopy confirmed terminal ileum intussuscepted into the caecal wall, which could not be reduced laparoscopically. Conversion to open laparotomy was performed, with right hemicolectomy and side-to-side ileocolic stapled anastomosis (Figs 3 and 4). The patient was discharged on postoperative day 9 without complications.

Histopathology revealed a polypoid tumour (8 × 6 × 2.5 cm) at the ileocolic junction invading to the muscularis propria, with both margins clear. Microscopy showed sheets of atypical lymphoid cells with medium-to-large nuclei, vesicular chromatin, and prominent nucleoli. Immunohistochemistry confirmed B-cell non-Hodgkin lymphoma: CD20+, BCL2+, BCL6+, MUM1+, and c-MYC+; negative for CD3, Synaptophysin, and Cyclin D1. The patient was referred to oncology and commenced chemotherapy. At one-month follow-up he was clinically well.

## Discussion

This case illustrates several clinically important features of adult ileocolic intussusception. First, the presentation was atypical and protracted — a pattern well recognized in adult intussusception, where symptoms are often chronic and non-specific [4]. In our patient, ASD added a further diagnostic barrier, as impaired communication prevented accurate symptom reporting. Clinicians should maintain a high index of suspicion for surgical pathology in patients with ASD who present with behavioural change or vague abdominal complaints.

Second, the lead point was B-cell non-Hodgkin lymphoma — an unusual but recognized cause of adult intussusception [7]. Primary intestinal lymphoma typically presents with non-specific abdominal symptoms and is often diagnosed only at surgery or on histopathological examination of the resected specimen. The immunohistochemical profile in this case (CD20+, BCL2+, BCL6+, MUM1+, and c-MYC+) is consistent with a high-grade phenotype requiring systemic chemotherapy.

Third, CT was critical to diagnosis and operative planning, demonstrating the extent of intussusception and characterizing the lead-point lesion preoperatively [5]. Given that ~40% of adult intussusception lead points are malignant, right hemicolectomy with adequate lymphadenectomy was the appropriate operative strategy, adhering to oncological principles [6]. Attempted manual reduction without resection would risk perforation and lymph node under-staging.

## Conclusion

Adult ileocolic intussusception caused by lymphoma can present insidiously, particularly in patients with impaired communication. Early cross-sectional imaging, prompt surgical resection with oncological margins and multidisciplinary postoperative care are essential to optimize outcomes.
